# A Case Report of Malignant Cerebellopontine Angle Lesion Highlighting the Interdisciplinary Diagnostic Challenge in the Case of Unilateral Progressive Hearing Loss

**DOI:** 10.3390/jcm13123483

**Published:** 2024-06-14

**Authors:** Riccardo Marzolino, Veronica Castro, Valeria Gambacorta, Eleonora Tonon, Elisabetta Cattaruzzi, Eva Orzan

**Affiliations:** 1 Otorhinolaryngology and Audiology Unit, Institute for Maternal and Child Health—IRCCS “Burlo Garofolo”, Via dell’Istria 63, 34137 Trieste, Italy; 2 Section of Otorhinolaryngology, Department of Medicine and Surgery, University of Perugia, 06129 Perugia, Italy; 3 Pediatric Radiology Department, Institute for Maternal and Child Health IRCCS “Burlo Garofolo,” 34137 Trieste, Italy

**Keywords:** unilateral hearing loss, cerebellopontine angle lesion, B-cell acute lymphoblastic leukemia

## Abstract

The authors present the case of a young boy who experienced progressive unilateral hearing loss initially believed to be unrelated to any other medical condition. **Methods:** The patient received a thorough evaluation, which included a comprehensive battery of audiological tests, a CT scan, and a gadolinium-enhanced MRI. **Results:** A repeated imaging investigation revealed the presence of a mass that mimicked a vestibular schwannoma (VS), but despite this, the boy was ultimately diagnosed with cerebral manifestations of B-cell acute lymphoblastic leukemia (B-ALL). **Conclusions:** Cerebral lesions originating from the internal auditory canal are rare in cases of B-ALL. In this case, the initial signs and symptoms of the disease were solely related to the audiovestibular system, making the diagnostic process particularly complicated. Unilateral hearing loss cases may indicate the presence of potentially life-threatening conditions, even if the hearing loss appears to be clinically non-syndromic. For these reasons, unilateral hearing losses necessitate a comprehensive interdisciplinary diagnostic approach from the very start of auditory manifestation and, in particular, if the hearing impairment demonstrates threshold progression.

## 1. Case Report

A 12-year-old male patient was initially admitted to the Department of Otolaryngology and Audiology of the Institute for Maternal and Child Health—IRCCS “Burlo Garofolo” for a second opinion, regarding a left hearing loss that presumably arose within the last year. The patient had previously undergone a gadolinium-free MRI, and the reports indicated a negative result.

**Figure 1:**
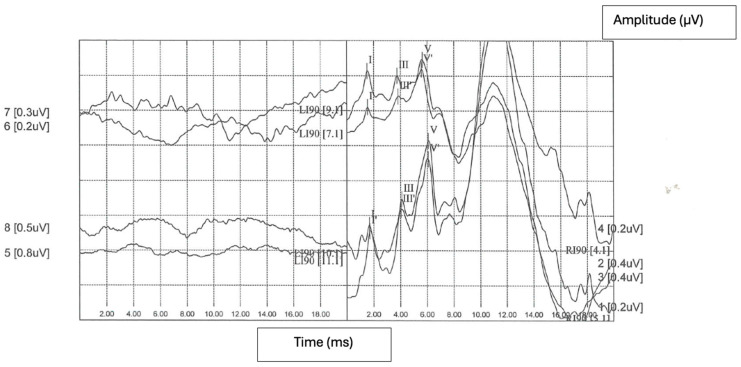
Click-evoked ABR revealing normal morphology and latencies on the right side but absent on the left side. The Roman numerals (I, III, V) correspond to the peaks of the ABR waves.

During the comprehensive medical history assessment, he denied blurring, facial weakness or numbness, dysphagia, dysphonia, or vertigo. He had recently felt sometimes imbalanced but had not noted ataxia or difficulty completing his normal tasks. His past medical history was silent; it did not reveal any audiological risk factors. He underwent a complete examination that revealed a normal external auditory canals and tympanic membranes. Facial nerve function, the remaining cranial nerves, and neurologic examination were all normal. A comprehensive set of audiological tests were performed: the audiogram revealed left-sided anacusis; tympanograms were bilaterally normal; the ipsilateral and contralateral acoustic reflexes configuration was representative for unilateral left sensorineural hearing loss; distortion product otoacoustic emissions (DPOAEs) were elicited on the right but not on the left side; a click-evoked auditory brainstem response (ABR) revealed normal morphology and latencies on the right side but they were absent on the left side (Figure 1). The vestibular evaluation demonstrated left vestibular deficit. 

**Figure 2:**
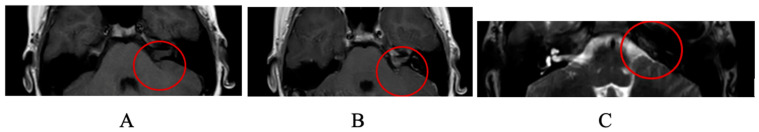
(**A**–**C**): The first MRI-obtained scan demonstrating the enhancing features of the lesion (highlighted by a red circle): (**A**) (T1 sequence), (**B**) (T1 sequence after gadolinium), and (**C**) (T2 sequence).

Overall, the audiological examinations were consistent in indicating the presence of a profound left sensorineural hearing loss and uncompensated left vestibular deficit. An in-depth analysis of the previously conducted magnetic resonance imaging revealed the presence of a neoformation within the left internal auditory canal (IAC). A CT scan was conducted, followed by a gadolinium-enhanced MRI. The results of the MRI conducted by our Institute indicated that the lesion had increased in size (measuring 13.8 × 5 × 6 mm) and revealed the presence of another lesion at the cerebellopontine angle (CPA). For this additional lesion, a schwannoma of the facial nerve was supposed (Figure 2A–C).

**Figure 3:**
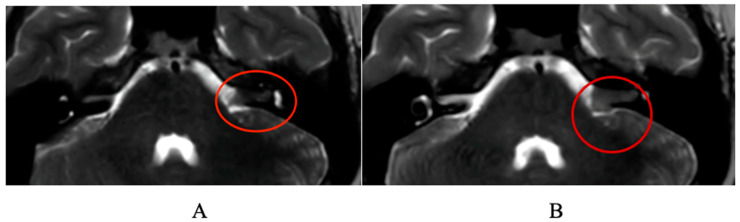
Comparison of the two MRI-obtained scans showing the increase in the lesion (highlighted by a red circle) from 13.8 × 5 × 6 mm (**A**) to 17 × 5 × 10 mm (**B**).

Genetic tests were requested to identify mutations related to neurofibromatosis and schwannomatosis genes. Over the next months, the boy developed two episodes of facial paralysis, responsive to corticosteroid therapy with complete recovery. Therefore, a further hospitalization was performed. Because of reported tiredness and back pain, a blood count was conducted, which yielded normal results. A further cerebral MRI and a back MRI were performed; the cerebellopontine angle (CPA) tumor grew up to 17 × 5 × 10 mm (Figure 3A,B), and a further dorsal lesion, compatible with dorsal neurinoma/schwannoma, was evidenced.

Meanwhile, urgently requested genetic analyses did not reveal any pathogenic mutations linked to the neurofibromatosis and schwannomatosis genes. 

A few weeks later, a sudden onset of fever, headache diplopia, nausea, vertigo, bradycardia, and drowsiness required hospitalization with an urgent CT scan which ruled out life-threatening problems. A complete blood count was requested, revealing thrombocytopenia along with a lymphoblast population that accounted for 10% of the peripheral blood. This finding confirmed the diagnosis of B-cell acute lymphoblastic leukemia (B-ALL). 

The intracranial leukemic mass at the CPA-IAC level was confirmed by the diminution of the lesion after starting systemic therapy for ALL. In fact, the MRI conducted after chemotherapy showed a significant decrease in the size of the lesions. However, although the mass decreased in size, there was no concurrent threshold improvement, thus confirming the presence of left anacusia on the audiometric examination conducted after the systemic therapy.

## 2. Discussion

Although unilateral sensorineural hearing loss (USNHL) is relatively common [1], it is often underdiagnosed and underappreciated in children and adolescents [2]. The prevalence of unilateral SNHL in children is estimated to be approximately 1.7 per 1000 live births, which rises up to 3% at school age [1]. In approximately 20–30% of patients, progression occurs [3]. In recent years, there have been several attempts to draw up guidelines or recommendations regarding the diagnostic process of USNHL [4]. The usual approach is that diagnostic tests are guided by clinical information. If history taking and physical examination offer no clue, the first diagnostic steps are testing for congenital cytomegalovirus (CMV) infection and performing imaging because of the high frequency of abnormalities [5]. Laboratory testing and genetic screening are not routinely performed, and very limited studies regarding progressive USNHL and its outcome are available. Gupta et al. conducted a prospective study involving 40 patients who had idiopathic unilateral sudden sensorineural hearing loss (SNHL). They discovered a significant correlation between the distribution of random blood sugar levels and the improvement of patients after therapy for sudden SNHL (*p* = 0.045). Nevertheless, there was no notable correlation observed between the treatment outcome in sudden sensorineural hearing loss (SNHL) and other blood parameters. Additionally, the severity of progressive SNHL did not show any connection to other blood parameters. The authors highlighted the lack of literature on the topic of “Unilateral Idiopathic Progressive Sensorineural Hearing Loss” in relation to its correlation with the nature of the disease. They suggested that future studies should be conducted to investigate the progressive variant of the disease and its outcomes [6]. Inner ear radiologic abnormalities are frequently observed in children with sensorineural hearing loss (SNHL), and their detection has a direct influence on prognosis and treatment decisions. Consequently, numerous writers recommend that all children with sensorineural hearing loss (SNHL) have radiologic imaging as a component of their assessment [7]. CT and MRI are utilized to assess sensorineural hearing loss (SNHL). Computed tomography is a highly effective method for detecting abnormalities in the bone labyrinth and middle ear. Magnetic resonance imaging (MRI) has a higher soft-tissue resolution and provides superior visualization of the internal structures of the cochlea, surpassing computed tomography (CT) in its ability to evaluate the cochleovestibular and facial nerves within the internal auditory canal, the cerebellopontine angle cistern, and any abnormalities along the auditory pathway and central nervous system [8]. Brain MR imaging with and without gadolinium is often used in patients with SSHL to exclude causes of cochlear and retrocochlear hearing loss such as other cerebellopontine tumors, brain stem infarctions, and demyelinating disease [9]. Radiologists must therefore familiarize themselves with the relevant anatomic variants and the diverse array of pathologic conditions that can be seen on these MRI scans [10]. Historically, contrast-enhanced MRI was considered the “gold standard” as a screening examination in patients with ASNHL prior to the advent of high-resolution 3D sequences [11]. More recent studies have suggested that high-resolution 3D T2WI alone are able to detect even small IAC lesions and that post-contrast sequences are unlikely to contribute clinically relevant information [12,13], while also reducing the acquisition time and cost of screening and follow-up MRIs, as well as avoiding patient exposure to gadolinium (evidence demonstrating cumulative sequestration and deposition of gadolinium in bone and brain tissue). In cases where a non-contrast screening MRI demonstrates a filling defect that could potentially be indicative of a retrocochlear pathology, further characterization with contrast-enhanced MRI would be necessary to confirm the presence of a tumor and to exclude other causes of fluid loss [11].

There is currently no agreement on the best imaging method for unilateral sudden sensorineural hearing loss (USNHL). Some experts suggest using CT scans as the primary option, while others argue for a greater emphasis on MRI or even using both methods together [14].

In their review and meta-analysis, Fabienne G. Ropers et al. found that imaging techniques were able to identify the cause of hearing loss in approximately 35% to 37% of cases. However, none of these findings had any direct therapeutic implications. Instead, imaging primarily provided information about the prognosis and hereditary factors in a small subset of children, specifically those with enlarged vestibular aqueduct. According to these experts, there are currently insufficient data to strongly advocate imaging for children with unilateral sensorineural hearing loss (USNHL). When making decisions together, it is important to thoroughly consider the benefits and risks of imaging [15]. In contrast, Muzzi et al. [16] highlighted the importance of early computed tomography (CT) scans for diagnosing unilateral sensorineural hearing loss. Inner ear malformations (IEMs) have been linked to unilateral sensorineural hearing loss (USHL) and an elevated susceptibility to bacterial meningitis in children. Timely identification of the specific type of IEM is essential as CSF leakage through the IEM can lead to recurring bacterial meningitis, particularly in cases of incomplete partition type 1 or common cavity abnormalities [17]. Temporal bone CT scans can detect inner ear malformations in cases of unilateral sensorineural hearing loss. It is considered unacceptable to postpone the detection of cerebrospinal fluid (CSF) leak through IEM after repeated episodes of meningitis during the Universal Newborn Hearing Screening (UNHS) period. It is recommended to perform an early CT scan to assess for unilateral sudden hearing loss (USHL) and consider preventive surgery for individuals at a high risk of cerebrospinal fluid (CSF) leak [16].

Paul et al. conducted a retrospective analysis of eighty children with USNHL, of whom 19% exhibited progression and 7.5% became bilateral. MRI should be the gold standard, as cochlear nerve deficiency is common, and CMV infection screening should be thorough. Genetic etiologies appear to differ from bilateral HL. Further genetic study in this area is required [5].

In our case, the disease presented solely auditory signs and symptoms, whereas the radiological scans were not entirely conclusive. The lack of early hematological indications also made the differential diagnosis especially challenging. The rapid evolution and the appearance of sudden suggestive symptoms allowed the definitive diagnosis, which would otherwise have necessitated a biopsy of the lesions.

Before contemplating an idiopathic cause in the presence of sudden unilateral sensorineural hearing loss, it is necessary to investigate the underlying causes and consider potential differential diagnoses.

The first aspect that requires analysis in clinical cases of progressive unilateral hearing loss is certainly the careful and accurate observation of the radiological examinations. The radiologist’s initial assumption was vestibular schwannoma, which immediately raised concerns about neurofibromatosis. Indeed, the clinical features and the localization of the lesion was suggestive of a vestibular schwannoma (VS), but typical radiological signs of a VS were not completely present. Typically, VS lesions appear hyperintense on T2 [18], while the current lesion appeared hypointense on T2. Conversely, the imaging features that indicated a diagnosis of vestibular schwannoma (VS) included the following: the lesion exhibited enhancement after the administration of contrast and the widening of the internal auditory canal [18]. The second lesion highlighted on MRI, expressing the same radiological features, given the extension to the geniculate ganglion, raised doubts about the presence of a contextual facial nerve schwannoma (FNS). The presence of two apparent schwannomatous lesions in this patient led to the hypothesis of neurofibromatosis. Neurofibromatosis is a genetic disease that affects the nervous system and causes the formation of benign tumors called neurofibromas [19]. There are three main types of neurofibromatosis: NF1, NF2, and schwannomatosis.

Hematological disorders can be included in the differential diagnoses for sudden sensorineural unilateral hearing loss. Approximately 25% of leukemia patients may encounter ear complications, typically characterized by moderate and unilateral symptoms [20]. Furthermore, earlier reports have commonly shown bilateral involvement in leukemic patients who experienced sensorineural hearing loss [21]. The link between leukemia and hearing loss is not a new discovery. It was first noted by Donne [22] and subsequently by Vidal [23] around the time Virchow defined leukemia as a distinct clinical condition in the mid-19th century. Since then, numerous clinical and histopathological reports have addressed hearing loss in leukemia patients. The initial frequency of otologic symptoms in leukemia patients was reported by Druss [24] to be 16.5%, but subsequent studies have shown much higher rates [20,25,26]. However, these reports encompass not only sensorineural hearing loss but also other otologic issues such as conductive hearing loss, vertigo, facial palsy, and ear infections. The incidence of sensorineural hearing loss specifically linked to leukemia is thought to be significantly lower. Although there have been occasional case reports describing acute sensorineural hearing loss as the first symptom of leukemia, such instances are quite rare [21,27,28,29,30]. In our case, the leukemia associated with acute sensorineural hearing loss was B-cell acute lymphocytic leukemia. Paparella et al. [20] noted that otologic symptoms were most common in patients with acute lymphocytic leukemia. However, the specific type of leukemia may not be a significant factor, as there have been reports of acute sensorineural hearing loss occurring as the initial symptom in cases of chronic myeloid leukemia [27,29,30] and chronic lymphocytic leukemia [28]. Additionally, acute sensorineural hearing loss can also be caused by other hematologic disorders and conditions, such as Waldenstrom’s macroglobulinemia [31] and severe hemorrhagic diathesis [32]. Since Politzer’s first histological description in 1885 [33], numerous studies have examined histopathological changes in the middle and internal ear of leukemia patients. These changes fall into three categories: (1) hemorrhage, (2) leukemic infiltration, and (3) infection [20]. Reviewing cases where inner ear hemorrhage was the primary temporal bone pathology shows that acute hearing loss in these instances occurred just days before death [34,35,36]. Therefore, cochlear hemorrhage seems to be the leading cause of acute sensorineural hearing loss in leukemia patients [37]. Leukemic infiltration is another potential cause of acute hearing loss [27], though it frequently coincides with inner ear hemorrhage [38,39]. Infections are commonly found in the middle ear but not in the inner ear [6]. Previous histopathologic reports have occasionally noted fibrosis and new bone formation in the inner ear, likely as a late result of hemorrhage [37]. However, these changes could also be sequelae of an inner ear infection when inflammation is seen in the middle ear. Hyperviscosity syndrome from hyperleukocytosis is another possible cause of sensorineural hearing loss, though it typically requires leukocyte counts above 500,000/mm^3^ [40]. Disseminated intravascular coagulation (DIC), as seen in our case, may damage the inner ear not only through hemorrhage but also by obstructing small inner ear vessels via intravascular coagulation.

The diagnosis of CNS leukemia is usually confirmed by the presence of leukemic blasts in CSF after lumbar puncture. MRI findings in CNS leukemia include meningeal and nerve root enhancement, thickening and abnormal enhancement of the optic nerve, retinal hemorrhage, and dura-based focal masses [23]. On MR images, the lesions are T2 hyperintense compared with white matter and have restricted diffusion and homogeneous enhancement after contrast administration [41]. Also, in our case, the lesion exhibited enhancement after the administration of contrast. In our case, the lesions highlighted were probably a direct infiltration of the CNS by leukemic cells; in fact, both their characteristics on the MRI and their reduction in size following the administration of the chemotherapy (that which did not occur after the administration of corticosteroids) were suggesting that the lesions were therefore primary leukemic infiltration instead of hemorrhage, edema, or infection [20]. Three previous pediatric cases have reported intracranial leukemic masses as the initial manifestation of B-ALL19 in the literature. These cases include a 2-year-old girl with a large lesion at the epidural level, a 13-year-old girl with a lesion localized in the frontal lobe at the cerebral level, and an 11-year-old girl with multiple brain injuries [42]. A variety of treatment strategies have been employed for intracranial leukemic deposits. Surgical debulking has been used with a number of patients, with varying results, and may help to rapidly improve associated neurologic symptoms. Radiation has also been described in combination with chemotherapy and sometimes surgery and may help with rapid reduction in the size of the mass; however, it is associated with adverse long term side effects. Chemotherapy, both systemic and intrathecal, remains the gold standard of treatment [42]. Our patient underwent chemotherapy treatment in a timely manner. Although the MRI showed a decrease in the masses, no improvement in auditory symptoms were observed.

## 3. Conclusions

Since hearing deterioration is the most common localizing symptom of CPA lesions, contrast-enhanced MRI is urgent and mandatory, particularly in cases of USNHL that arise and show signs of progression.

Cerebral manifestations of B-ALL leukemia before hematological evidence are rare. Furthermore, B-ALL cerebral lesions arising from the internal auditory canal are extremely rare. To our knowledge, no previous similar cases have been reported in the literature. In this case, all the initial signs and symptoms of the disease were exclusively auditory, with the absence of early hematological signs, making the differential diagnosis extremely problematic.

Given the substantial differences in treatment and prognosis between benign and malignant lesions, this case highlights the great importance of interdisciplinary diagnostic efforts in the case of unilateral hearing loss associated with growing IAC/CPA lesion at a young age.

## Data Availability

Data are contained within the article.
